# Early-stage spatial disease surveillance of novel SARS-CoV-2 variants of concern in Germany with crowdsourced data

**DOI:** 10.1038/s41598-021-04573-1

**Published:** 2022-01-18

**Authors:** Timo Mitze, Johannes Rode

**Affiliations:** 1 Department of Economics, University of Southern Denmark, Campusvej 55, 5230 Odense, Denmark; 2 Faculty of Law and Economics, Technische Universität Darmstadt, Hochschulstraße 1, 64289 Darmstadt, Germany

**Keywords:** Epidemiology, Statistics, Viral infection

## Abstract

The emergence and rapid spread of novel variants of concern (VOC) of the coronavirus 2 constitute a major challenge for spatial disease surveillance. We explore the possibility to use close to real-time crowdsourced data on reported VOC cases (mainly the Alpha variant) at the local area level in Germany. The aim is to use these data for early-stage estimates of the statistical association between VOC reporting and the overall COVID-19 epidemiological development. For the first weeks in 2021 after international importation of VOC to Germany, our findings point to significant increases of up to 35–40% in the 7-day incidence rate and the hospitalization rate in regions with confirmed VOC cases compared to those without such cases. This is in line with simultaneously produced international evidence. We evaluate the sensitivity of our estimates to sampling errors associated with the collection of crowdsourced data. Overall, we find no statistical evidence for an over- or underestimation of effects once we account for differences in data representativeness at the regional level. This points to the potential use of crowdsourced data for spatial disease surveillance, local outbreak monitoring and public health decisions if no other data on new virus developments are available.

## Introduction

When novel variants of concern (VOC) of the severe acute respiratory syndrome coronavirus type 2 (SARS-CoV-2) were first reported in late 2020, scientists, policy makers and the general public soon asked essential questions: Do these VOC find it easier to transmit from human to human compared to previously circulating strains? Do VOC lead to faster disease progressions, more severe disease cases and higher fatality rates? How can VOC spread be effectively monitored at different spatial levels to respond to local outbreaks and mitigate viral spread?

Early studies for the UK, where the Alpha variant (B.1.1.7 lineage) of SARS-CoV-2 was first noted in mid-November 2020, found an approximately 50% higher human-to-human transmissibility compared to the wild type of the virus^1–4^. Similar evidence was provided for the Beta variant (B.1.351 lineage) first detected in South Africa and the Gamma (P.1 lineage) variant^4,5^. Studies on a higher human-to-human-transmissibility of VOC were complemented by evidence on increased disease severity, fatality rates^6–8^ and a larger risk of international disease transmission^9^.

In Germany, the first case of a SARS-CoV-2 infection based on the Alpha variant was reported on December 24, 2020. The Alpha variant soon became the dominant source of SARS-CoV-2 infections in Germany reaching a share of more than 50% within the first 2 months of 2021 and over 90% within the first 3 months of 2021 in all SARS-CoV-2 infections (calculated on the basis of genome data from the Global Initiative On Sharing All Influenza Data (GISAID) hCoV-19 Tracking of Variants project^10^; similar findings for the testing site of the Charité-Universitätsmedizin Berlin are reported in^11^). The rapid spread of the Alpha variant constituted a main triggering factor for the third infection wave in Germany in spring 2021 and led to strict nationwide shutdown measures^12^.

Given the significance of VOC for overall SARS-CoV-2 infection dynamics, the goal of this study is twofold: First, it shall document our approach to provide near-time statistical estimates of spatial disease trends in Germany associated with the emergence and rapid spread of VOC during the first weeks in 2021. Novel to our approach is the use of crowdsourced data on reported VOC cases for statistical analyses at the local area level in Germany. We combine these data with administrative data on SARS-CoV-2 cases and hospitalized cases (intensive care patients with and without artificial ventilation). The second goal is to assess the quality of crowdsourced VOC data in retrospect and to evaluate its prospective use as an available close to real-time source for the analysis of epidemiological indicators during the SARS-CoV-2 pandemic.

The provision of close to real-time (near-time) estimates is hugely important to monitor local disease outbreaks and to uncover population level trends at different levels of spatial aggregation. We focus on two key indicators here: (1) the 7-day incidence rate, i.e., the number of newly reported SARS-CoV-2 infections in the last 7 days per 100,000 population, and (2) the hospitalization rate, i.e., the number of hospitalized COVID-19 patients in intensive care per 100,000 population. While the 7-day incidence rate was the main indicator used for disease surveillance and public health decisions in Germany in early 2021, the hospitalization rate is used here as an important stress indicator for local health care systems. While the incidence rate can be confounded by different regional test intensities, the hospitalization rate does not suffer from this bias.

## Spatial disease surveillance with crowdsourced data

In early 2021, national and local health authorities in Germany faced the problem that data on the spatial spread of novel VOC were largely missing. While the Robert Koch Institute (RKI), the central institution for disease surveillance in Germany, had started to monitor VOC spread early on in 2021, available data were limited in two dimensions. On the one hand, the RKI utilized data on whole genome sequencing, which, however, were only available on the basis of ad-hoc sampling procedures supplied by collaborating laboratories^13^ and it was unclear, how representative sampling was at the regional level.

On the other hand, administrative data on confirmed VOC cases supplied by local health authorities according to the German Infection Protection Act (GIPA) were initially incomplete and reported summaries only covered broad regional aggregates, i.e., the German federal states (NUTS-1 level). For instance, in its first report on the state of VOC spread in Germany dated February 5, 2021, the RKI documented that a total of 168 cases of the Alpha variant had been reported across federal states until the end of calendar week (CW) 4^13^. One month later, on March 3, 2021, in its third report, the RKI revised this number to 1556 cases just in CW 4^14^.

These revisions partly reflect an unavoidable testing lag, i.e., the time needed to identify a VOC case from patient data (by genome sequencing or variant-specific PCR testing). Another complicating factor in early 2021 was that not all local health authorities in Germany, which are in charge of collecting information on SARS-CoV-2 cases according to the GIPA, were yet connected to Germany’s digital surveillance system^14^. While this made disease surveillance at the regional level already very difficult, the lack of comprehensive data on reported VOC cases below the level of federal states rendered a comprehensive local-area outbreak monitoring almost impossible.

We propose the use of crowdsourced data collected from news paper reports and social media as a possibility to fill this data gap and to monitor VOC spread at the local area level (NUTS-3 level) in Germany. By combining crowdsourced VOC data with public health data on the overall infection dynamics, it is possible to provide early-stage estimates of the human-to-human transmissibility and case severity of VOC relative to the wild type of the SARS-CoV-2 virus. This is done here by correlating space-time differences in the detection of VOC cases and overall SARS-CoV-2 epidemiological trends. Statistical estimations are conducted as near-time predictions covering the period November 15, 2020, to February 4, 2021 during which the share of the Alpha variant increased from zero to approximately $40\,\%$ of all reported SARS-CoV-2 cases (according to GISAID data for Germany).

Crowdsourced VOC data are taken from the virus variant-tracking project by^15^. The crowdsourcing project has started in January 2021 as a private, non-profit initiative to collect and publish close to real-time information on confirmed VOC cases covering the Alpha, Beta and Gamma variants of the SARS-CoV-2 virus. Individual case documentation is based on newspaper reports, social media entries and other available public health reports at the local level. The project received attention from the broader public through nation-wide media coverage (e.g.,^16,17^). For our near-time estimates, we have extracted and processed data until February 4, 2021. As it had been argued previously, crowdsourced data can prove to be of paramount importance in monitoring and controlling the spread of infectious diseases^18^.

An earlier application of crowdsourced data for an epidemiological analysis of SARS-CoV-2 is documented in^19^ based on approx. 500 disease cases in China during January 2020. The authors use case data from social media and newspaper reports to estimate several key epidemiological parameters including the relative infection risk by age groups and the mean incubation date, and the time between first symptoms and hospitalization. While such information is of tremendous importance for disease surveillance, it is stressed in^20^ that the use of crowdsourcing data raises a number of challenges from a statistical point of view, thereby potentially biasing estimation and hypothesis testing. Specifically, the accuracy of a crowdsourcing exercise needs to be checked against the presence of sampling and non-sampling errors^21^.

While sampling errors, i.e., differences between the selected sample and the underlying population, are one source for estimation biases when using crowdsourced data, non-sampling errors may arise, e.g., due to wrong interpretations of the data by the collectors, self-selection of collectors and—in the case of spatial data—locational errors. We have checked for non-sampling errors ex ante during the data extraction phase by running consistency checks for approx. 10% of the individual entries covered in the crowdsourcing project. We additionally assess the role of sampling errors for the robustness of our results in an ex-post manner. This may provide further insights into and recommendations for the general use of crowdsourced data for spatial disease surveillance beyond the specific application presented here.

## Results

### Near-time estimates of epidemiological trends associated with VOC spread

**Figure 1:**
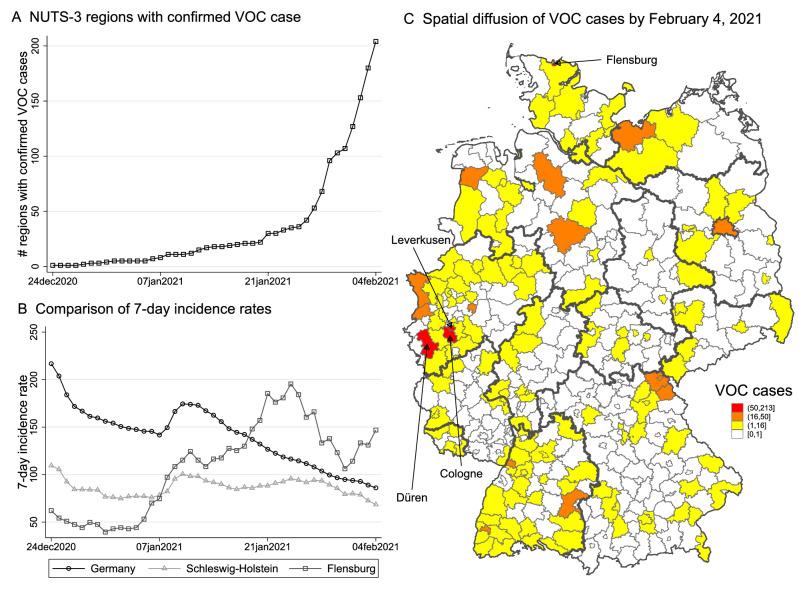
Temporal and spatial distribution of VOC cases in Germany. Panel (**A**) counts NUTS-3 regions with confirmed VOC cases over time. Panel (**B**) compares the 7-day incidence rate (by day of reporting) for Germany, Schleswig-Holstein, and Flensburg. Panel (**C**) shows the spatial spread of VOC cases by February 4, 2021. Flensburg and Cologne, Düren and Leverkusen report most confirmed cases.

According to the processed crowdsourced data, by February 4, 2021, in 204 out of 401 NUTS-3 regions at least one confirmed case of a SARS-CoV-2 infection with one of the three VOC (Alpha, Beta, Gamma) had been reported and the number of affected regions grew exponentially by the end of January (see Panel A of Fig. 1). Out of all recorded cases in the crowdsourcing project by that date, approx. 88% dated from the Alpha variant, 12% from the Beta variant and $<1$% from the Gamma variant. Panel C of Fig. 1 displays the spatial distribution of cumulative VOC cases summed over all three variants. The map shows that the city of Flensburg and a cluster of three NUTS-3 regions (Cologne, Leverkusen and Düren) in North-Rhine Westphalia (NRW) were among the top 5% of most affected regions in Germany by that time.

A first inspection of the incidence rate development in these regions around the timing of VOC emergence has nourished our concerns that variant spread significantly drove local infection dynamics. In Flensburg, a 90,000-inhabitant city in Schleswig-Holstein, the 7-day incidence rate drastically increased relative to the average development in Schleswig-Holstein and Germany throughout January 2021 (see Panel B of Fig. 1). According to local health authorities in Flensburg, new infections mainly happened at illegal parties, i.e., against prevailing lockdown rules, on New Year’s Eve. A link between these parties and VOC-based infections was established on January 24, 2021. By February 4, the number of confirmed VOC cases had already grown to 146. In the three NRW cities, first VOC cases were reported between January 12 and January 23, 2021.

To further investigate the link between VOC detection and the overall infection dynamics across German NUTS-3 districts, we employ two commonly used indicators for disease surveillance: (1) the 7-day incidence rate, i.e., the number of newly reported SARS-CoV-2 infections in the last 7 days per 100,000 population, and (2) the hospitalization rate, i.e., the number of hospitalized COVID-19 patients in intensive care per 100,000 population. We first apply Synthetic Control Method (SCM) estimation for case study analysis of Flensburg and the three NUTS-3 regions in NRW. This is followed by static and dynamic panel estimates (Difference-in-Difference estimation, Panel Event Study) for all 401 German districts. We give a brief method description below and details in the Supplementary Appendix.

#### Synthetic control method (SCM)

**Figure 2:**
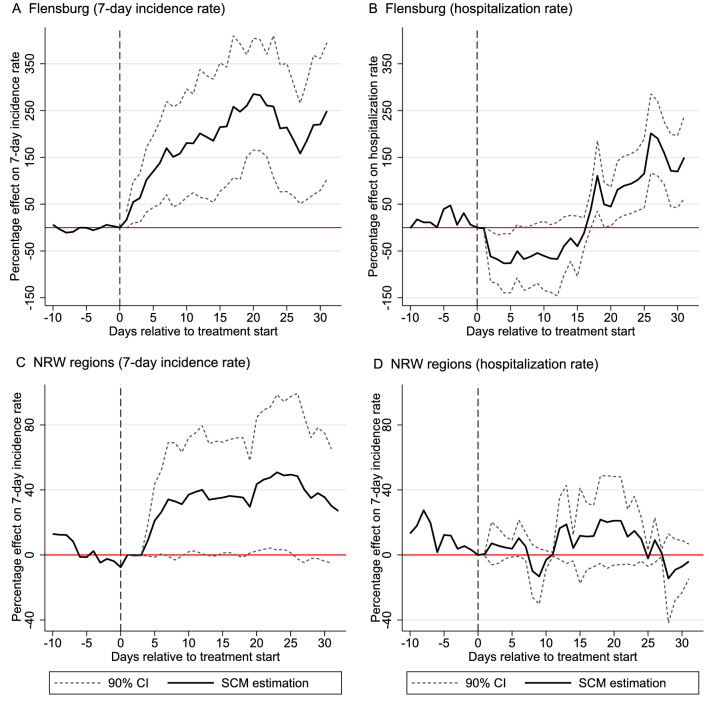
SCM estimates for the relative percentage increase in the 7-day incidence rate for Flensburg (Panel **A**) and NRW regions (Panel **B**) and the hospitalization rate for Flensburg (Panel **C**) and NRW regions (Panel **D**) vis-à-vis their synthetic control groups. Treatment start is set to January 5, 2021 in all cases. 90% confidence intervals are constructed on the basis of pseudo *P* values (see “Methods” section for details).

In Fig. 2, we estimate the change in incidence and hospitalization rates for Flensburg (Panel A and B) and the cluster of three NRW cities (Panel C and D) around the timing of the first VOC reporting. The figure displays the estimated percentage effect on these rates relative to their last pre-treatment values together with 90% confidence intervals (calculated from pseudo *P* values) to assess the statistical significance of the estimated changes in incidence rates. We set the start of the treatment period in the SCM analyses to January 5, 2021, which is at least one week before the confirmation of the first VOC cases in all four treated regions. Because we use SARS-CoV-2 infection data recorded by symptom onset, we argue that starting the treatment period at least one week before the first confirmed VOC case is sufficient to account for incubation times^22^. Hospitalization rates have previously been estimated to lag the infection development (onset of symptoms) by about 4–8 days^23^.

Evaluated against the counterfactual infection development in the synthetic control group of similar regions without confirmed VOC case, the observed 7-day incidence rate for Flensburg becomes significantly larger after treatment start (see Panel A of Fig. 2). No significant differences between treated and the synthetic control group are observed for the time period prior to January 5. With regard to effect size, VOC spreading in Flensburg is associated with a tripling of the incidence rate (20 days after treatment start) compared to the counterfactual situation of not having experienced VOC spread, mainly through illegal New Year’s celebration parties.

Consistent with prior evidence on the delay between infection and hospitalization, the hospitalization rate for Flensburg shown in Panel C of Fig. 2 shows a significant increase approx. 16 days after treatment start. Although the estimated percentage change shows a doubling to tripling of hospitalized patients in intensive care 30 days after treatment start, we need to consider that this effect is calculated on the basis of a relatively small absolute number of hospitalized patients per 100,000 population at the local area level (i.e., 2 patients per 100,000 population in the last pre-treatment observation and, on average, 8 additional cases per 100,000 population in Flensburg during the treatment period).

The SCM results for the NRW cluster furthermore indicate that Flensburg may be a very exceptional case since illegal parties have likely served as super-spreading events for VOC infections. Treatment effect estimates for the NRW cluster report an increase in the 7-day incidence rate by approx. 40% (after 20 days) and no significant increase in hospitalization rates until February 4, 2021.

#### Difference-in-difference (DiD) estimation

Table 1 reports DiD estimation results, which compare the 7-day incidence rates in all NUTS-3 regions with and without confirmed VOC cases before and after the first VOC reporting in a region. To increase estimation power, data on the three VOC types have been pooled because the literature indicates similarly higher human-to-human transmissibility of these VOC compared to the wild type of the virus^1–5^. Further, in terms of health policy decisions, it is most important to identify potential higher human-to-human transmissibility and less which variant is responsible for it. Still, our results mainly refer to the Alpha variant, which accounts for 88% of cases reported in our sample of the crowdsourcing project. In Table 1, we report results for different time windows: in the baseline specification, we set treatment start to the date of the first VOC reporting in the region; we then extend the treatment period to 7, 14, and 21 days before this date to capture latent transmissions from an already imported but yet undetected VOC case.

**Table 1:** Difference-in-difference estimations for association between first reporting of variants of concern (VOC) and 7-day incidence rates at the local area level in Germany.

Treatment start	(1)	(2)	(3)	(4)
	Day first reporting	7 days before	14 days before	21 days before
**Panel A: Baseline**				
VOC Reporting	13.14$$^{**}$$	7.63	3.61	0.08
	[4.2,22.1]	[$-1.7$,16.9]	[$-5.7$,12.9]	[$-9.0$,9.2]
N	22441	22441	22441	22441
NUTS-3 regions	401	401	401	401
**Panel B: First VOC reported before January 22, 2021**				
VOC Reporting	15.09$^{+}$	17.83$^{*}$	17.74$^{*}$	13.65$^{+}$
	[$-1.7$,31.9]	[2.7,32.9]	[2.6,32.8]	[$-1.1$,28.4]
N	12712	12712	12712	12712
NUTS-3 regions	227	227	227	227
**Panel C: Top-10% regions in terms of VOC count**				
VOC Reporting	35.00$$^{**}$$	30.65$$^{**}$$	24.63$$^{**}$$	17.61$$^{**}$$
	[17.9,52.1]	[17.3,44.0]	[11.6,37.7]	[4.8,30.4]
N	13552	13552	13552	13552
NUTS-3 regions	242	242	242	242
**The following applies to all panels**				
Fixed Effects & Controls	Yes	Yes	Yes	Yes

Across columns, the dependent variable is the 7-day incidence rate in a NUTS-3 region at a given day. We always consider the time period between November 15, 2020 and February 4, 2021. In Panel A, we take into account all 401 NUTS-3 regions in Germany. By February 4, 2021, 204 NUTS-3 regions reported a VOC case and 197 did not (Panel B: 30 NUTS-3 regions with at least one reported VOC case before January 22, 2021; Panel C: 45 NUTS-3 regions belonging to the top 10% of sample regions in terms of VOC count). In Panel A, *VOC Reporting* is a dummy variable defined as one if a VOC case has been reported in a NUTS-3 region, else zero (Panel B: *VOC Reporting* is one if a VOC case has been reported before January 22; Panel C, *VOC Reporting* is one if a regions belongs to the top-10% regions in terms of VOC count). Some observations have been dropped because we lack information on some control variables (e.g., on Daily mobility change in relation to 2019 for December 4–7, 2020). We include NUTS-3 and day fixed effects and a linear and a squared trend for four different NUTS-3 region types. We also control for a 1 day, a 7-days and a 14-days lag of the following variables: first, the reported SARS-CoV-2 cases within the previous 7 days, second, the reported SARS-CoV-2 cases within the previous 7 days in neighboring NUTS-3 regions, third, the daily mean temperature at 2 m above ground in $$^{\circ }\hbox {C}$$, and fourth, the daily mobility change in relation to 2019. We include (but do not show) a constant in all regressions. 95% confidence interval based on clustered *SE* (on NUTS-2 level) in parentheses; $$^{+}$$ $$p<0.1$$, $$^{*}$$ $$p<0.05$$, $$^{**}$$ $$p<0.01$$.

We find a significant statistical trend associated with VOC reporting at the local area level. For the overall sample with at least one confirmed VOC case, the estimates in Panel A point to an average increase in the 7-day incidence rate by 13.14 [95% CI 4.2, 22.1] cases per 100,000 population or approx. 12% (evaluated against the average 7-day incidence rate in comparative regions of 111). While effects are of similar size for the sub-sample estimates limiting treated regions to those with a VOC case confirmed before January 22 in Panel B (i.e., early treated regions for which we observe at least 14 days of treatment), we find a remarkably higher effect size for the subsample defining the treatment groups as belonging to the top 10% (90th percentile) of absolute VOC counts. For the latter, we find an increase in the incidence rate of 32% on average (35.00 [CI 17.9, 52.1] additional cases per 100,000 population). Generally, the extension of treatment periods by 7, 14 and 21 days ahead of the first VOC results in smaller point estimates in all three panels (with declining statistical significance). This indicates that infection trends appear to change around the date of first VOC reporting and not significantly before that date.

Confounding factors may challenge our estimates. We control for several potential confounders in the link between first VOC reporting and the 7-day incidence rate. These include daily mobility patterns and daily weather data at the regional level, time-invariant region-specific factors (through NUTS-3 level fixed effects), day-specific factors that affect all regions in the same way (through day fixed effects), spatial spillovers in local SARS-CoV-2 infection numbers by calculating spatial lag variables capturing the SARS-CoV-2 development in the geographical neighborhood of each NUTS-3 regions. The “Methods” section and the SI appendix contain further details and source descriptions.

Another major concern is that the estimated correlations between VOC reporting and the increase in the 7-day incidence rate as shown in Table 1 are simply a reflex of a change to higher testing intensities in regions with confirmed VOC cases, which may then raise the number detected but potentially asymptomatic SARS-CoV-2 infections. To rule out this effect, we report DiD estimations results for the hospitalization rate as outcome variable in Table 2. We show estimates for the baseline specifications in analogy to Table 1 with treatment start being set to the day of first VOC reporting in a region.

**Table 2:** Difference-in-difference estimations for association between first reporting of variants of concern and number of COVID-19 patients in intensive care at the local area level in Germany.

	(1)	(2)	(3)
	Baseline	First VOC reported before Jan22, 2021	Top-10% regions in terms of VOC count
**Panel A: All patients in intensive care**			
VOC reporting	0.37	0.50	1.30$$^{**}$$
	[$-0.1$,0.9]	[$-0.5$,1.5]	[0.5,2.1]
N	22217	12656	13496
NUTS-3 regions	397	226	241
**Panel B: Patients with artificial ventilation**			
VOC reporting	0.14	$$-0.05$$	0.71$$^{**}$$
	[$-0.2$,0.5]	[$-0.8$,0.7]	[0.2,1.2]
N	22217	12656	13496
NUTS-3 regions	397	226	241
**The following applies to all panels**			
Fixed effects & Controls	Yes	Yes	Yes

In Panel A, across columns, the dependent variable is the number of COVID-19 patients in intensive care in a NUTS-3 region at a given day. Panel B is similar but refers to patients in intensive care with artificial ventilation. We always consider the time period between November 15, 2020 and February 4, 2021. In Panel A, we report results for 397 NUTS-3 regions in Germany, for which we have daily data on the number of patients in intensive care. By February 4, 2021, 204 NUTS-3 regions reported a VOC case and 197 did not (Panel B: 30 NUTS-3 regions with at least one reported VOC case before January 22, 2021; Panel C: 45 NUTS-3 regions belonging to the top 10% of sample regions in terms of VOC count). In Panel A, *VOC Reporting* is a dummy variable defined as one if a VOC case has been reported in a NUTS-3 region, else zero (Panel B: *VOC Reporting* is one if a VOC case has been reported before January 22; Panel C, *VOC Reporting* is one if a regions belongs to the top-10% regions in terms of VOC count). Some observations have been dropped because we lack information on some control variables (e.g., on Daily mobility change in relation to 2019 for December 4–7, 2020). We include NUTS-3 and day fixed effects and a linear and a squared trend for four different NUTS-3 region types. We also control for a 1 day, a 7-days and a 14-days lag of the following variables: first, the reported SARS-CoV-2 cases within the previous 7 days, second, the reported SARS-CoV-2 cases within the previous 7 days in neighboring NUTS-3 regions, third, the daily mean temperature at 2 m above ground in $$^{\circ }\hbox {C}$$, and fourth, the daily mobility change in relation to 2019. We include (but do not show) a constant in all regressions. 95% confidence interval based on clustered *SE* (on NUTS-2 level) in parentheses; $$^{+}$$ $$p<0.1$$, $$^{*}$$ $$p<0.05$$, $$^{**}$$ $$p<0.01$$.

While the DiD results point to positive but insignificant correlations for the sample of all regions in column (1) and the subsample of regions with an early VOC reporting before January 22 in column (2), we find a statistically significant increase in the hospitalization rate for regions in the top10% (90th percentile) of reported VOC cases in column (3). The estimated increase of 1.30 [CI 0.5, 2.1] additional COVID-19 patients in intensive care per 100,000 population translates into a 42% rise compared to the average hospitalization rate in comparison regions (3.08 patients in intensive care per 100,000 population). Panel B illustrates that the results are robust to using the number of COVID-19 patients in intensive care with artificial ventilation per 100,000 population as the dependent variable.

#### Panel event study (PES)

**Figure 3:**
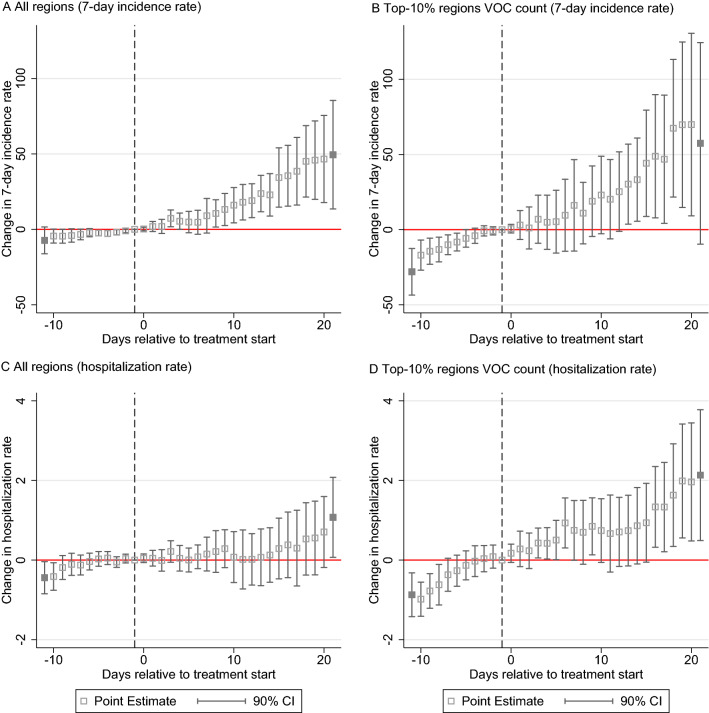
Panel event study estimates for trend development in the 7-day incidence rate (Panel A and B) and the hospitalization rate (Panel C and D) in regions with confirmed VOC cases around the day of first VOC reporting. Hollow squares show estimated daily point estimates; grey squares report cumulative estimates beyond the maximum number of leads (10) and lags (20) around the start of the first VOC reporting in treated regions. The dashed vertical line indicates that the last day before treatment start (− 1) serves as benchmark period for the estimated daily lead and lag coefficients.

We use PES to investigate dynamic effects over time. Figure 3 reports daily estimates for the 7-day incidence rate (Panel A and B) and the hospitalization rate (Panel C and D) for the overall sample of regions and the subsample of treated regions belonging to the top 10% (90th percentile) of VOC cases. Effects are calculated for the relative time before and after VOC reporting in each region. Panel A shows for the sample of all regions with at least one VOC case that 20 days after VOC reporting the 7-day incidence rate has increased by approx. 48% compared to the average incidence rate in treated regions and the last pre-treatment observation. Panel A indicates 50 additional SARS-CoV-2 infections per 100,000 population 20 days after treatment start relative to an sample average to 105 infections per 100,000 population in the last pre-treatment period.

For regions in the top 10% (90th percentile) of VOC cases, we find in Panel B of Fig. 3 that the cumulative effect grows even stronger during this 20-day time window. Regional effect heterogeneity increases as well (as indicated by the widened confidence intervals). The fact that effects turn statistically insignificant beyond the 20-day time window may hint at mitigating effects from strict containment measures implemented in some of these regions.

With regard to the hospitalization rate, significant effects can be observed approx. 15 days after treatment start for the sample of treated regions belonging to the top 10% in terms of reported VOC cases. After 20 days, the hospitalization rate is estimated to be approx. 38% higher in treated regions compared to their last pre-treatment observation. This corresponds with two additional patients in intensive care per 100,000 in treated regions evaluated against the average of 5.19 patients in intensive care per 100,000 population before treatment start. Generally, Panel A to Panel D in Fig. 3 clearly indicate that incidence and hospitalization rates in regions with VOC tend to grow over time relative to the counterfactual situation. The effects visualized in Fig. 3 do not give indication for positive early anticipation effects from latent confounding events in treated regions determining the observed trend development. Our analysis thus indicates that the growth in incidence and hospitalization rates strongly coincides with the reporting of VOC cases at the local area level.

### Ex-post validity checks of crowdsourced VOC data and near-time estimates

**Figure 4:**
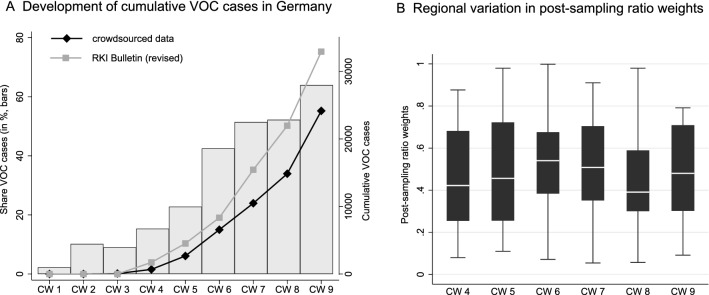
Comparison of cumulative VOC counts covered by different data sources in the first calendar weeks (CW) 2021. Panel (**A**) shows time-series for the development of cumulative VOC cases in Germany reported by the virus variant-tracking crowdsourcing project by^15^ and those reported by German health authorities and documented in^14,24,25^ later on. The time period shown covers the first 9 calendar weeks in 2021 (January 4 to March 7, 2021). Bars in Panel (**A**) measure the percentage share of VOC cases (Alpha, Beta and Gamma variants, jointly) in all submitted genomes for Germany to the Global Initiative On Sharing All Influenza Data (GISAID) hCoV-19Tracking of Variants project. Data are obtained from^10^. Panel (**B**) shows box plots for the regional distribution of post-sampling ratio (PSR) weights by calendar weeks. Details on the calculation of PSR weights exploiting differences between VOC cases covered in the crowdsourced database and the RKI bulletins for individual German federal states as shown in this figure are given in the main text.

The accuracy and reliability of treatment effect estimates depends on the underlying data quality. As a first ex-post measure for the coverage and representativeness of the crowdsourced VOC data, Panel A of Fig. 4 plots the cumulative development of VOC cases identified in the close to real-time virus variant-tracking crowdsourcing project by^15^ with revised epidemiological data from German health authorities published by^14,24,25^ with a delay of several weeks. We take the revised data from German health authorities as the best available approximation for the unknown true distribution of VOC cases in Germany. Both time series closely follow a common trend, with a widening gap only after calendar week 6 (February 15, 2021). The Pearson’s correlation coefficient measuring the fit between the cumulative development of VOC cases is $\rho =0.78$ (and 0.58 for newly reported VOC cases by calendar week). This points to a high coverage of all publicly reported VOC cases in the virus variant-tracking crowdsourcing project during the time period when the share of VOC in all SARS-CoV-2 cases increased significantly, i.e., our sample period until February 4, 2021.

**Figure 5:**
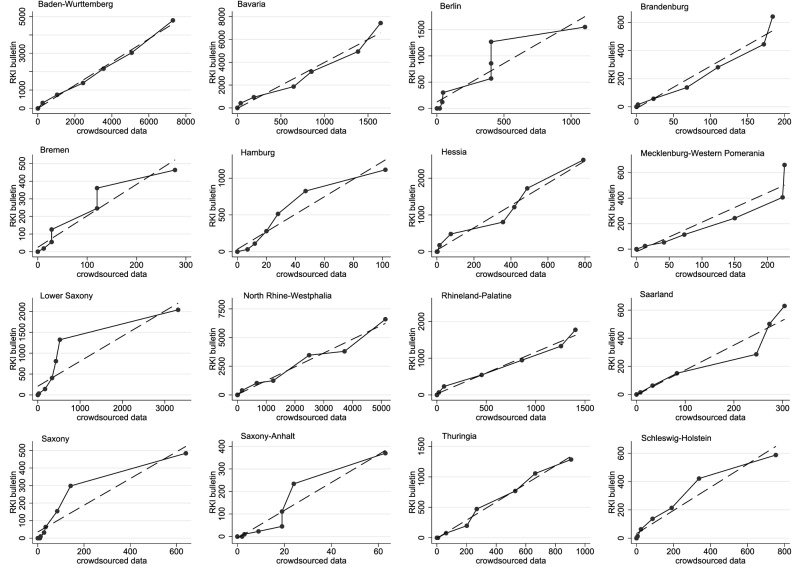
Regional variation of VOC coverage in crowdsourced data. The individual panels correlate the cumulative development of VOC cases covered in the close to real-time virus variant-tracking crowdsourcing project by^15^ to those reported by German health authorities later on and documented in^14,24,25^ at the German federal state (NUTS-1) level. Dashed lines in each panel indicate the linear fit between crowdsourced and RKI data.

Figure 5 shows the pairwise correlation between covered VOC cases across both datasets at the German federal states level. These regional comparisons are used to calculate sample weights utilizing the concept of a post-sampling ratio as suggested in^20^. Based on the argument that the revised RKI data provide the best available benchmark value for the overall population of VOC cases, we calculate the post-sampling ratio (PSR) as the ratio of cumulative VOC cases covered in the crowdsourced and the RKI data (by federal states and calendar weeks). Since both an over- and under-reporting is a potential source for a sampling error in the crowdsourced data, the post-sampling ratio is used to calculate sample weights as a proximity measure based on the absolute difference of PSR values from one, i.e., the case when crowdsourced and RKI data exactly match each other. The regional distribution of PSR weights ranging between 0 and 1 is shown in Panel B of Fig. 4 by calendar week. Values closer to 1 indicate a higher representativeness of the crowdsourced data.

Since no weekly RKI data are available before calendar week 4, we use time averages of PSR weights by federal states to evaluate the robustness of the reported DiD estimates in Table 1. The logic of PSR weighting is that NUTS-3 regions located in a federal state (NUTS-1 level) with a more representative data coverage receive a higher sample weight in the re-estimation exercise. The results in Table 3 report the difference in estimated VOC coefficients between the original (Ordinary Least Squares, OLS) and PSR-based Weighted Least Squares (WLS) estimates for the DiD model. The difference is calculated for both standard PSR weights as well as squared weights to further reduce the sample weight of regions with low data representativeness. A positive difference points to an overestimation of effects in the original OLS regressions. We also report results of a *z*-test for coefficient equality^26^.

**Table 3:** VOC effect differences and tests for equality of regression coefficients between original near-time and weighted estimates using post-sampling ratio weights.

	(1)	(2)	(3)
	Baseline	Reported before Jan 22, 2021	Top-10% regions in terms of VOC count
**Panel A: Standard PSR weights**			
7-day Incidence rate	$-\,0.85$	$-\,1.83$	3.88
*z* Value	(0.48)	(0.24)	(0.50)
Hospitalization rate	$-\,0.05$	$-\,0.04$	0.09
*z* Value	(0.04)	(0.03)	(0.07)
Hospitalization rate (with art. ventilation)	$-\,0.03$	$-\,0.03$	0.05
*z* Value	(0.02)	(0.02)	(0.14)
**Panel B: Squared PSR weights**			
7-day Incidence rate	$-\,1.11$	$-\,2.87$	7.19
*z* Value	(0.91)	(0.38)	(0.98)
Hospitalization rate	$-\,0.04$	$-\,0.04$	0.21
*z* Value	(0.12)	(0.06)	(0.48)
Hospitalization rate (with art. ventilation)	$-\,0.03$	$-\,0.04$	0.10
*z* Value	(0.09)	(0.10)	(0.28)

Results report the difference in the estimated VOC coefficients $diff=\left( \beta _{original}-\beta _{reweighted}\right)$ between the original near-time estimates reported in Tables 1 and 2 and the re-weighted specification using post-sampling ratio weights (and their squared values) for the 7-day incidence rate, the hospitalization rate (all patients in intensive care) and the hospitalization rate for patients with artificial ventilation. The reported *z* Values are the resulting test statistic from a *z*-test for coefficient equality across different regression models defined as $z = \left( \frac{\beta _{original}-\beta _{reweighted}}{\sqrt{(SE\beta _{original})^2 + (SE\beta _{reweighted})^2}}\right)$, where $SE\beta _{i}$ is the standard error of $\beta _{i}$^26,27^. A statistically significant positive coefficient difference would point to an overestimation of the association between VOC reporting and the development of an epidemiological indicator at the local area level in Germany for the crowdsourced VOC data. Both the original and the re-weighted estimation specification use the same set of regressors as reported in the footnotes of Tables 1 and 2 and in the SI appendix.

**Figure 6:**
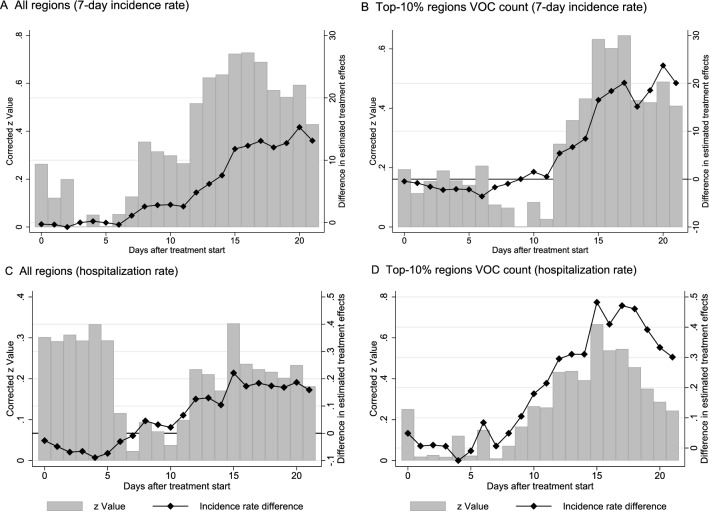
Difference in estimated daily PES coefficients and statistical tests for coefficient equality across models. The time-series plot in Panel (**A**) shows the development in the coefficient difference for the dynamic effect of VOC reporting on the 7-day incidence rate as $\left( \beta _{original}-\beta _{reweighted}\right)$, where $\beta _{original}$ are the daily PES coefficients from Panel A of Fig. 3 and $\beta _{reweighted}$ are the corresponding re-estimated coefficients using post-sampling ratio weights. Both specifications follow the estimation setup described in the footnote of Fig. 3 and Panel A plots coefficient differences for lags (20) around the start of the first VOC reporting in a treated regions. Bars report the *z* Values obtained from a *z*-test of coefficient equality across specification as described in the notes of Table 3. Panels (**B**) shows coefficient differences for the effect on the 7-day incidence rate and corresponding *z* Values for the subsample of treated regions belonging to the top 10% in terms of VOC count. Panels (**C**,**D**) report the corresponding overall and subsample results for the hospitalization rate (all patients in intensive care).

In no case, the test rejects coefficient equality between the original and weighted estimates. However, for the subsample of regions belonging to the top-10% of regions in terms of VOC count, the results in Table 3 point to a tendency of the original estimates to produce larger effects. This tendency is also seen in Fig. 6, which shows the difference in estimated daily treatment effects for the PES analysis originally reported in Table 3. Although we, again, cannot reject coefficient equality on the basis of a series of *z*-tests, Fig. 6 points to a growing difference between OLS and WLS estimates over time, i.e., a longer time lag between first VOC reporting and observed values for the 7-day incidence and hospitalization rate at the local level. Carefully speaking, our original estimates from crowdsourced data, which do not correct for sampling errors, may show a tendency to report higher effect uncertainty for longer prediction horizons and potentially overestimate effect size. This result is obviously linked to our data structure, which covers fewer NUTS-3 regions in the treatment group once we increase the time lag between first VOC reporting and epidemiological outcomes in the sample period.

## Discussion

Main goals of this paper were, firstly, to describe the initial regional dynamics of VOC spread (predominately the Alpha variant) within a country after international importation using novel crowdsourced data, secondly, to provide near-time estimates of epidemiological trends associated with VOC reporting at the local area level in Germany, and, thirdly, to evaluate the robustness of the crowdsourced data for statistical analyses of spatial disease surveillance. In line with earlier studies, mainly for the UK, we find that the 7-day incidence rate increased by around 30% in regions with confirmed VOC cases compared to regions without such a case after the first reporting. Moreover, a higher number of reported VOC cases at the local area is found to increase the incidence rate dynamically over time. Our estimates also point to a positive correlation between VOC reporting and regional hospitalization rates. When we evaluate data representativeness ex post, we, firstly, find a high correlation between reported VOC cases from the utilized crowdsourcing database and revised data from local health authorities in Germany, published by the RKI. Secondly, we do not find statistical evidence that underlying sampling errors affect the robustness of our original near-time estimates using crowdsourced data.

## Limitations

Despite the fact that we do not find evidence for estimation biases as result of sampling errors, our results are limited in several important dimensions. A first complicating factor is that we cannot directly control for SARS-CoV-2 *test intensities* at the regional level. Test intensity may be higher in regions with confirmed VOC relative to comparison regions. Our results may partly capture this effect if more testing leads to a higher number of detected SARS-CoV-2 infections with only mild or no symptoms. To preclude a bias in the estimation results, we have controlled for a battery of fixed effects. Further, we have analyzed the development of hospitalization rates at the local area level next to the 7-day incidence rate because the hospitalization rate is not affected by the testing regime. Our findings also confirm a significant correlation between VOC reporting and the number of hospitalized patients per 100,000 population in those regions that have a relatively high number of VOC cases (top 10%). We find that VOC importation to these regions is associated with an increase in the hospitalization rate by 40%. Also, the number of patients with artificial ventilation rises. This points to future potential threats for local health systems in regions with a high VOC spread.

We would also like to stress that we *estimate the correlation* between confirmed VOC cases and the 7-day incidence rate of infections or the hospitalization rate. While our results provide robust evidence against the statistical null hypothesis of no association between the presence of VOC and the development of local infection and hospitalization rates, we cannot infer the causal nature of this relationship. I.e., using data at the population level for NUTS-3 regions only allows us to identify correlations between VOC spread and epidemiological trends, but no causal statements can be made whether VOC have a higher transmissibility than previously circulating virus strains and whether VOC infections lead to more severe COVID-19 disease cases. Representative genomic data at the local area level may help to comprehensively answer this question^28–30^. In cases where such data are unavailable, however, we argue that crowdsourcing projects together with carefully executed near-time estimates of statistical trends may inform public health authorities about the need for actions to mitigate the local and global transmission of VOC at an early stage^31^.

Our analyses may also be subject to *ecological fallacy* because we do not observe individual level information but aggregates on the regional level. If no individual-level information is available, studies on the regional population-level are best-practise (for similar approaches, e.g.,^19,32–35^).

Finally, we focus on *one specific crowdsourcing project* with many contributors, which also received wide media attention and thus attracted many collaborators. Other crowdsourcing projects may not deliver information of similar reliability. Of course, it lies within the responsibility of those, who want to exploit information from crowdsourcing projects, to carefully check the data for sampling and non-sampling errors.

## Methods

We employ complementary statistical tools to estimate correlations between the timing of VOC reporting, local incidence rates and hospitalization rates. Given the unequal spatial distribution of confirmed cases and the emergence of local clusters with relatively high VOC counts (as shown in Panel C of Fig. 1), we start out with comparative case studies for most severely affected regions, namely Flensburg and the cluster of three NUTS-3 regions (Cologne, Leverkusen and Düren). Estimations are conducted by the synthetic control method (SCM) for single and multiple treated units, which compares the infection development in case study regions to the development in a synthetic control group composed of similar regions without reported VOC cases^36,37^.

To establish a meaningful counterfactual situation, the SCM approach constructs the synthetic control group as the weighted average of regions in the donor pool, i.e., all regions without reported VOC case. Weights are estimated through a minimum distance approach for predictor values in the pre-treatment period. We provide an overview of predictors used for SCM estimation together with a description of SCM implementation and statistical inference in the SI appendix. While SCM offers favorable features for detailed case study analyses related to COVID-19 research^32,34,38^, its focus on case study regions limits its ability to provide general estimates of infection trends associated with reporting of VOC cases in Germany.

We therefore complement SCM estimation by difference-in-difference (DiD) regressions and a panel event study (PES). A review of the suitability of the DiD approach for COVID-related empirical research is given in^39^. DiD estimation aims at identifying differences in epidemiological trends (time differences) across regions with and without treatment (cross-sectional difference), where treatment is defined as the reporting of at least one (or multiple) VOC case(s) at the regional level. The complementary PES allows to control for staggered treatment start and offers insights into treatment effect dynamics^40^. Given the dynamic nature of COVID-related epidemiological data and the staggered nature of events, e.g. policy actions to mitigate disease spread, PES has been frequently applied in several COVID-related studies^33,35,41,42^.

We provide an extended method section in the SI appendix including a description of identification challenges and how we approach them. To ensure comparison across estimation methods, we apply all three approaches to the same set of outcome variables, i.e. the 7-day incidence rate and the number of hospitalized patients in intensive care (with and without artificial ventilation) per 100,000 population. Data on daily SARS-CoV-2 infections (by the onset of symptoms) for each of the 401 NUTS-3 regions have been collected from the COVID-19 dashboard of the Robert Koch Institute (RKI), which is in charge for disease surveillance in Germany^43^. The number of hospitalized patients in intensive care for 397 NUTS-3 regions with an own hospital is taken from the INFAS corona database^44^. We account for several confounding factors in the link between the treatment indicator and the evolution of the outcome variable. These include daily mobility patterns and daily weather data at the regional level together with time-invariant demographic structures of regions. We also account for spatial spillovers in local SARS-CoV-2 infection numbers by calculating spatial lag variables capturing the recent SARS-CoV-2 development in the geographical neighborhood of each NUTS-3 region. Our study focuses on the end of the year 2020 and early 2021. During this time period, the cumulative reported infection numbers were below 3% of total population on February 4, 2021. Further, vaccine availability was very limited across Germany with around 1% of total population vaccinated on February 4, 2021. Because vaccine availability was also comparatively homogeneous across German regions (and therefore captured by our day fixed effect), we do not have to control for the acquired immunity in the population in each region. The SI appendix contains data descriptions and source information.

### Ethical standards

All methods/data collection were performed in accordance with relevant guidelines and regulations.

## Supplementary Information

Supplementary Information.

## Data Availability

All study data and codes to replicate the estimation results (including robustness tests) are stored in a publicly available data repository accessible through the following DOI: 10.6084/m9.figshare.13946903.
