# Supplementary material for: Early-stage spatial disease surveillance of novel SARS-CoV-2 variants of concern in Germany with crowdsourced data
Source: Sci Rep. 2022 Jan 18;12:899. doi: 10.1038/s41598-021-04573-1 (PMC8766449; doi:10.1038/s41598-021-04573-1)
Supplement: Supplementary file 1 — Supplementary Information. [file 41598_2021_4573_MOESM1_ESM.pdf]

Supplementary Information for:

Early-stage spatial disease surveillance of novel SARS-CoV-2 variants of concern in Germany with crowdsourced data

Timo Mitze[1,*] **and Johannes Rode**[2]

[1]University of Southern Denmark, Department of Business and Economics, Campusvej 55, 5230 Odense, Denmark; ORCID: 0000-0003-3799-5200
[2]Technische Universität Darmstadt, Faculty of Law and Economics, Hochschulstraße 1, 64289 Darmstadt, Germany; ORCID: 0000-0002-3134-0759
[*]Corresponding author: tmitze@sam.sdu.dk

Extended Methods

Synthetic control method (SCM)

We use SCM to analyse two case studies for a single treated unit (Flensburg) and multiple treated units (cluster of three cities/regions [Cologne, Leverkusen and Düren] in North Rhine-Westphalia; all NUTS-3 level). In all four case study regions a SARS-CoV-2 variant of concern (VOC) has been confirmed by genome sequencing, which is used to identify treatment status. VOC are defined as the Alpha (B.1.1.7 lineage), the Beta (B.1.351 lineage) and the Gamma (P.1 lineage) SARS-CoV-2 variants. The objective of SCM is to compare the development in epidemiological outcome variables after treatment start in the two sets of treated region(s) vis-à-vis a synthetic control groups selected from a donor pool of 197 comparison regions without any confirmed VOC case during the entire sample period December 15, 2020 to February 4, 2021. Outcome variables are i) the 7-day incidence rate (SARS-CoV-2 infections per 100,000 population over the last seven days) and ii) the hospitalization rate (hospitalized patients in intensive care per 100,000 population). We compute daily treatment effects for a maximum of 31 days and express them as percentage difference to the last pre-treatment observation of the scaled outcome variable (to 100), see Fig. 2 in the main text.

We motivate the use of SCM as one element of our empirical identification strategy because the estimation approach has been shown to be a flexible and robust estimation tool that has previously been applied to COVID-related research, for instance, to study the effect of face masks on SAR-CoV-2 infection numbers in Germany [1] and lockdown effectiveness for a counterfactual of Sweden [2] and the USA [3]. The key identification approach of SCM is to establish a counterfactual that mimics a situation in which the treatment in treated regions (here: the emergence of VOC cases) would not have taken place. This is implemented by means of creating a synthetic control group consisting of the donor pool of comparison regions and by comparing the outcomes of treated units and the synthetic control after the start of the treatment. The match between treated regions and the synthetic control group is done through a minimum distance approach for a set of predictor variables evaluated along their pre-treatment values for treated regions and those in the donor pool. This ensures that pre-treatment differences in trends of the outcome variable are leveled. A formal description of the estimation approach and the underlying assumptions for effect identification are given in [4, 5, 6].

For our purpose of estimating the epidemiological effects of emerging VOC in German regions, we adopt and extend the data and estimation setup applied in [1]. For both SCM applications, we set the start of the treatment period to January 5, 2021 and identify treatment effects of VOC throughout January and early February. In all four cases, the reporting of the first confirmed VOC case took place at least one week after the start of the treatment period (Cologne: January 12, 2021, Leverkusen: January 18, 2021, Düren: January 23, 2021, Flensburg: January 24,

2021). This time lag between the start of the treatment and the reporting of the first VOC case should ensure that latent transmission effects are captured in the estimation. For instance, in the case of Flensburg, VOC infections could be traced back to illegal parties on December 31, 2020 [7]. Considering a median incubation time of 5 days for SARS-CoV-2 infections [8], we can thus expect that first VOC effects become visible in the data from January 5, 2021 onwards. This is before the first VOC case was confirmed through genome sequencing on January 24, 2021 for Flensburg.

Data on reported SARS-CoV-2 infections are taken from the Robert Koch Institute [9]. For our empirical analysis we use aggregate case numbers for each NUTS-3 region and day tracked on the basis of symptom onset for individual cases rather than the reporting date by local health authorities. This allows us to estimate the transmission timing of SARS-CoV-2 at the regional population level more precisely. We aggregate the data across age groups. Data on confirmed cases of the three novel VOC (B.1.1.7., B.1.351, P.1) together with their reporting dates are gathered from a public crowd-sourcing project [10], which bases case documentation on newspaper and public health reports. We have cross-checked data consistency by retracing individual cases and their timing from the documented source information and have conducted additional online searches for selected cases.

A relevant concern against SARS-CoV-2 case numbers is that they may grow with growing test intensity. Test intensity may rise in regions in regions with confirmed VOC cases. To rule out this effect, we use the hospitalization rate as alternative outcome. Specifically, we use daily information on the number of COVID-19 patients in intensive care (with and without artificial ventilation) per 100,000 population [11].

In the specification of SCM estimation, we account for the autoregressive dynamics of infections and the hospitalization rate by including the 7-day incidence rate and the absolute number of cumulative SARS-CoV-2 infections during the last 3 weeks before treatment start as time-varying predictors. Other time-varying predictors are the average daily temperature for each region during the last 2 weeks and changes in average daily mobility during the last 2 weeks before treatment start. Changes in average daily mobility per region are measured relative to a 2019 (pre-COVID) benchmark period. We use data on daily temperatures from Deutscher Wetterdienst [12] and data on mobility changes from [13].

We further include time-constant cross-sectional predictors characterizing regional demographic structures and the regional health care system as in [1] based on data from the INKAR online database of the Federal Institute for Research on Building, Urban Affairs and Spatial Development [14]. We use the latest year available in the database, which is 2017. Employed cross-sectional predictor variables include population density (Population/km2), regional settlement structure (categorial dummy), the share of highly educated population (in %), the share of female in population (in %), the average age of female and male population (in years), old- and young-age dependency ratios (in %), the number of physicians per 10,000 of population and pharmacies per 100,000 of population.

We conduct all SCM estimations in STATA using the SYNTH [15] and SYNTH_RUNNER [16] packages. Confidence intervals (CIs) are calculated from one-sided pseudo p-values obtained on the basis of comprehensive placebo-in-space tests. The latter tests calculate pseudo-treatment effects for all regions in the donor pool treating each of the regions as if it would have received the treatment of a confirmed VOC case by or after January 5, 2021. One-sided pseudo p-values are then calculated of the share of placebo-treatment effects that are larger than the observed treatment effects for treated regions and thus indicate the probability that the increase in the number of SARS-CoV-2 infections was observed by chance given the distribution of pseudo-treatment effects in the donor pool. To account for differences in pre-treatment match quality of the pseudo-treatment effects, only donors with a good fit in the pre-treatment period are considered for inference. Specifically, we do not include placebo effects in the pool for inference if the match quality of the control region, measured in terms of the pre-treatment root mean squared prediction error (RMSPE), is greater than 10 times the match quality of the treated unit [6]. Based on the obtained pseudo p-values we calculate confidence intervals as described in [17].

Robustness.– We mainly perform robustness tests by changing the composition of the donor pool. First, we exclude donor regions that were selected in the baseline SCM estimation. The idea behind this analysis is to preclude unintended selection effects resulting from latent VOC transmissions captured in the overall infection dynamics of donor regions in the pre-treatment period. Second, we reduce the pool of donor regions to those NUTS-3 regions

which are located in the same federal state as the treated regions (Schleswig-Holstein for Flensburg and North Rhine-Westphalia for Cologne, Leverkusen and Düren). This approach should minimize differences in public health measures, which are mainly decided under the authority of individual federal states in Germany. While mentioned but not reported in the manuscript in detail, we include codes to run all robustness tests in the replication files.

Difference-in-difference estimation (DiD)

To investigate average and dynamic treatment effects for the entire group of treated regions with at least one confirmed VOC case, we additionally run a series of panel regressions in a DiD and Panel event study (PES) framework. The sample period for the panel regressions includes the time period between November 15, 2020 and February 4, 2021. This ensures that we cover all confirmed VOC cases (in the currently best possible way) together with a sufficient pre-treatment period for each region of at least 3 weeks. As for the case of SCM, we use the 7-day incidence rate and the hospitalization rate as key outcome variables. For the incidence rate we measure the timing of infections in terms of symptom onset rather than reporting by local health authorities. We also use the same set of time-varying predictor variables as described above; cross-sectional predictors for the set of NUTS-3 regions are not included as we account for NUTS-3 region fixed effects in the panel regressions.

Specifically, we run DiD regressions as two-way fixed effects model of the following general form

$$y_{i,t} = \delta VOC_{i,t} + X_{i,t-j}\Gamma + \sum_{r=1}^{R} \gamma_r \left(RegionType_{r(i)} \times trend\right) + \sum_{r=1}^{R} \psi_r \left(RegionType_{r(i)} \times trend^2\right) + \tau_t + \mu_i + \varepsilon_{i,t}. \quad (1)$$

In equation 1, $y_{i,t}$ is the epidemiological outcome of interest (7-day incidence rate, hospitalization rate) observed for NUTS-3 region i and day t. The variable $VOC_{i,t}$ is our main treatment indicator, which takes values of 1 from the day onwards for which the first VOC case was confirmed in the region. The coefficient δ measures the direction and strength of the correlation between VOC reporting and outcome variables. We additionally test for latent transmission effects prior to the first reporting of a VOC (given that genome sequencing to identify SARS-CoV-2 variants may take up to 2 weeks). This is done by moving forward the date when the treatment dummy $VOC_{i,t}$ takes values of 1 for treated regions by 7, by 14 and by 21 days, respectively. Importantly, these extended treatment specifications do not test for early anticipation effects caused by latent confounding factors (this is done in the Panel Event Study), but averages estimated effects over a longer treatment period to capture potential latent transmissions prior to the first VOC confirmation as, for instance, identified for Flensburg in the SCM estimations.

It is important to control for factors that potentially confound the link between VOC and the overall SARS-CoV-2 incidence rate at the regional level. The set of confounding factors, which we can directly account for, is included in the variable vector $X_{i,t-j}$. Specifically, similar to the SCM application, we control for the number of SARS-CoV-2 cases in region i during the last, the second last and the third last week. We also include a spatially lagged variable covering the number of SARS-CoV-2 cases in region i's (direct) spatial neighbors during the last, the second last and the third last week. A spatial lag is important because infections can easily spread from one region to another region nearby, e.g., due to commuting or general mobility [18]. Spatial association between regions is measured through first-order contiguity, i.e. whether regions share a common border or not. We row standardize the resulting spatial weights matrix.

Further, we control for the average temperature [19] and the relative change in average daily mobility at $t-1$, at $t-7$ and at $t-14$ in region i. Controlling for mobility is important because lower mobility can be disease mitigating [20, 21, 22]. Including mobility effectively controls for lockdown measures implemented during the sample period and how people follow the rules. In addition, we include linear and quadratic time trends for four different region types $\left(RegionType_{r(i)}\right)$ classified on the basis of the region's settlement structure including region type 1 (large district-free cities, kreisfreie Städte), type 2 (urban regions, Landkreise), type 3 (rural regions, Landkreise) and type 4 (sparsely populated regions, Landkreise), i.e. $R = 4$. The classification of region types follows the definition of the Federal Institute for Research on Building, Urban Affairs and Spatial Development [14]. Table 1 shows descriptive statistics.

Table 1. Descriptive statistics DiD and Event Study.

	Mean	Std. Dev.	Min.	Max.
Panel A: Baseline with 7-day incidence rates				
7-day incidence rate	147	85	8	714
VOC Reporting	.07	.26	0	1
Reported SARS-CoV-2 cases within the previous seven days	296	399	5	8042
Spatially Lagged reported SARS-CoV-2 cases within the previous seven days	108	199	0	2778
Mean temperature at 2 m above ground in °C	1.8	3.5	-11	14
Daily mobility change in relation to 2019	-15	17	-73	76
N	22441			
Panel B: Panel B: Baseline with Hospitalization				
Number of COVID-19 patients in intensive care per capita times 100,000	6	6	0	68
Number of COVID-19 patients in intensive care with artificial ventilation per capita times 100,000	3.2	3.5	0	34
N	22217			

Notes: Observations are for each NUTS-3 region per day. We always consider the time period between November 15, 2020 and February 4, 2021. In Panel A, we take into account all 401 NUTS-3 regions in Germany. By February 4, 2021, 204 NUTS-3 regions reported a VOC case and 197 did not. In Panel B, we take into account 397 NUTS-3 regions in Germany, for which we have daily data on the number of patients in intensive care. By February 4, 2021, 201 NUTS-3 regions reported a VOC case and 196 did not. Some observations have been dropped because we lack information on some control variables (e.g., on Daily mobility change in relation to 2019 for December 4-7, 2020).

τ_t are time fixed effects for each day in the sample, which for instance control for daily changes in infection levels similar across regions. μ_i controls for time-constant region fixed effects, which could, e.g., be caused by region-specific SARS-CoV-2 testing intensities. $\varepsilon_{i,t}$ is the model's error term. We cluster standard errors at the NUTS-2 level (each of the 401 NUTS-3 regions belongs to one of the 38 NUTS-2 regions in Germany). We estimate δ and Γ using the REGHDFE package [23] in STATA, which allows us to control for τ_t, μ_i, γ_r and ψ_r.

Robustness.– Besides the full sample covering all treated regions, we also conduct estimations with sub-samples. First, we focus on those experiencing treatment early on (before January 22, 2021) to observe at least 14 days of treatment after the first confirmed VOC case for each treated region. Second, we study regions with a relatively high number of VOC reported cases. Here, we restrict the treated regions to the top-10 percentile of VOC counts, which corresponds to at least 9 VOC cases per region. The idea of this subsample is to test for treatment effect difference associated with confirmed VOC counts rather than the presence of at least one VOC case. Finally, we run estimations by variant type. In a first sub-sample, we focus on the British variant but allow for other reported variants. In a second sub-sample, we only consider the British variant. Similarly, we investigate effects for regions where the South-African variant was reported (together with potential other variants) and for regions for which only the South African variant was reported.

Panel event study (PES)

The estimation of the PES differs from the two-way fixed effects DiD specification mainly in the way that it accounts for the staggered emergence of a VOC in treated regions throughout the sample period. This allows us to identify dynamic treatment effects over time. Dynamic treatment effects may arise for different reasons: First, they could reflect early anticipation effects prior to the treatment start due to latent VOC transmissions or, second, they could result from other unobserved confounding factors systematically affecting incidence rates in treated regions around the treatment start. Thus, it is important to test for such early anticipation effects. The absence of statistically significant estimates for the latter but significant treatment effects could, accordingly, be interpreted in favor of our empirical identification strategy.

Moreover, we may expect that infection and hospitalizations effects do not immediately occur after the reporting of the first VOC case but potentially build up over time at the regional population level. This may particularly be the case if public health authorities can only imperfectly trace and mitigate VOC-related disease spread. In this case, the estimation of average treatment effects on treated (ATTs) as shown in equation 1 may potentially underestimate the dynamics of SARS-CoV-2 infections and hospitalizations subject to confirmed VOC cases. By including sufficient lag and lead terms in the estimation framework for the timing of treatment start, we can identify dynamic treatment effects.

The staggered nature of treatment start in different treated regions can be incorporated into the panel regression approach by translating the model from a specification in absolute time t (as shown in equation 1) to a specification that measures time for each region relative to treatment start. Together with the recognition of potential heterogeneity in the strength of treatment effects over time, the PES setup allows us to precisely estimate the impact of the passage of a treatment (here: VOC reporting in a region) that occurs at different times in different spatial units. A more formal presentation of the PES approach together with estimation challenges is given in [24, 25] among others. Prior COVID-related PES applications have dealt, for instance, with the infection effects from school re-opening in Germany [26] and [27], university students traveling during the U.S. spring break [28] or mass protests from the Black Lives Matter movement [29].

In the implementation of the PES approach, we include the same set of covariates and fixed effects as in the case of the two-way fixed effects DiD estimation. We also cluster standard errors at the NUTS-2 level. We set the maximum number of pre-treatment leads to 10 days and the maximum number of lags after the treatment start to 20 days. Further effects from leads/lags before (after) this range are accumulated to a single coefficient. To allow for an easy comparison of the SCM and PES results, we express all reported effects as percentage change relative to the observed 7-day incidence rate in the last pre-treatment period.

Robustness.– Besides the full sample covering all treated regions, we estimate the effects for sub-samples of regions. First, we focus on those experiencing treatment early on (before January 22, 2021 to observe at least 14 days of treatment after the first confirmed VOC case). Estimation results for this sub-sample are only shown in the replication files. Second, we study regions with a relatively high number of VOC reported cases. We restrict the treated regions to the top-10 percentile of VOC counts, which corresponds to at least 9 VOC cases per region.

We conduct the PES estimations in STATA. The analysis builds on the EVENTDD package [30]. We document the full analyses in the replication files, particularly the mentioned robustness tests.

Data availability

All study data and codes to replicate the near-time estimation results (including robustness tests) and the ex-post validity checks are stored in a publicly available data repository accessible through the following DOI: 10.6084/m9.figshare.13946903.

References

1. Mitze, T., Kosfeld, R., Rode, J. & Wälde, K. Face masks considerably reduce COVID-19 cases in Germany. *Proc. Natl. Acad. Sci.* **117**, 32293–32301, DOI: https://doi.org/10.1073/pnas.2015954117 (2020).

2. Cho, S.-W. S. Quantifying the impact of nonpharmaceutical interventions during the COVID-19 outbreak: The case of Sweden. *The Econom. J.* **23**, 323–344, DOI: https://doi.org/10.1093/ectj/utaa025 (2020).

3. Friedson, A. I., McNichols, D., Sabia, J. J. & Dave, D. Shelter-in-place orders and public health: Evidence from California during the COVID-19 pandemic. *J. Policy Analysis Manag.* **40**, 258–283, DOI: https://doi.org/10.1002/pam.22267 (2021).

4. Abadie, A. & Gardeazabal, J. The economic costs of conflict: A case study of the Basque country. *Am. Econ. Rev.* **93**, 113–132, DOI: https://doi.org/10.1257/000282803321455188 (2003).

5. Abadie, A., Diamond, A. & Hainmueller, J. Synthetic control methods for comparative case studies: Estimating the effect of California's tobacco control program. *J. Am. Stat. Assoc.* **105**, 493–505, DOI: https://doi.org/10.1198/jasa.2009.ap08746 (2010).

6. Cavallo, E., Galiani, S., Noy, I. & Pantano, J. Catastrophic natural disasters and economic growth. *The Rev. Econ. Stat.* **95**, 1549–1561, DOI: https://doi.org/10.1162/REST_a_00413 (2013).

7. Ove, J. Hohe Corona-Zahlen offenbar Spätfolge illegaler Silvesterpartys, Der Nordschleswiger, Freitag, 12. Februar 2021. https://www.nordschleswiger.dk/de/suedschleswig/hohe-corona-zahlen-offenbar-spaetfolge-illegaler-silvesterpartys (2021). Accessed: February 12, 2021.

8. Lauer, S. A., Grantz, K. H., Bi, Q. & et al. The incubation period of coronavirus disease 2019 (COVID-19) from publicly reported confirmed cases: Estimation and application. *Annals Intern. Medicine* **172**, 577–582, DOI: https://doi.org/10.7326/M20-0504 (2020).

9. Robert Koch Institute. Covid-19 Infektionen, general website (NPGEO Corona Hub):. https://npgeo-corona-npgeo-de.hub.arcgis.com/ (2021). Accessed: February 8, 2021.

10. Römer, C. *et al.* Tracking B.1.1.7, B.1.351 und P.1 Nachweise in Deutschland. https://docs.google.com/spreadsheets/d/1G-2TAslg1KOOlHayxVd8WMEia2wln02j_OerBHZjpnA/ (2021). Accessed: February 8, 2021.

11. Institut für angewandte Sozialwissenschaft (INFAS), DIVI-Intensivregister. Corona-Datenplattform, Intensivstationen, general website:. https://www.corona-datenplattform.de/dataset/intensivstationen (2021). Accessed: Febraury 24, 2021.

12. Deutscher Wetterdienst (German Weather Service). Climate data center (cdc), general website:. https://www.dwd.de/EN/climate_environment/cdc/cdc.html (2021). Accessed: February 8, 2021.

13. Statistisches Bundesamt (Destatis). Experimental data, mobility indicators based on mobile phone data, daily change in mobility at administrative district level compared to the previous year (past 31 days to the current end are available online, older data available upon request at Statistisches Bundesamt), general website:. https://www.destatis.de/EN/Service/EXDAT/Datensaetze/mobility-indicators-mobilephone.html (2021). Accessed: February 8, 2021.

14. INKAR. Indikatoren und Karten zur Raum- und Stadtentwicklung: Bundesinstitut für Bau-, Stadt- und Raumforschung (Federal Institute for Research on Building, Urban Affairs and Spatial Development), general website:. https://www.inkar.de/ (2021). Accessed: June 4, 2020.

15. Abadie, A. Using Synthetic Controls: Feasibility, Data Requirements, and Methodological Aspects. *J. Econ. Lit.* **59**, 391–425, DOI: https://doi.org/10.1257/jel.20191450 (2021).

16. Galiani, S. & Quistorff, B. The synth_runner package: Utilities to automate synthetic control estimation using synth. *The Stata J.* **17**, 834–849, DOI: https://doi.org/10.1177/1536867X1801700404 (2017).

17. Altman, D. G. & Bland, J. M. How to obtain the confidence interval from a p value. *BMJ* **343**, DOI: https://doi.org/10.1136/bmj.d2090 (2011).

18. Kosfeld, R., Mitze, T., Rode, J. & Wälde, K. The covid-19 containment effects of public health measures: A spatial difference-in-differences approach. *J. Reg. Sci.* **61**, 799–825, DOI: https://doi.org/10.1111/jors.12536 (2021). https://onlinelibrary.wiley.com/doi/pdf/10.1111/jors.12536.

19. Baker, R. E., Yang, W., Vecchi, G. A., Metcalf, C. J. E. & Grenfell, B. T. Assessing the influence of climate on wintertime SARS-CoV-2 outbreaks. *Nat. Commun.* **12**, 846, DOI: https://doi.org/10.1038/s41467-021-20991-1 (2021).

20. Xiong, C., Hu, S., Yang, M., Luo, W. & Zhang, L. Mobile device data reveal the dynamics in a positive relationship between human mobility and COVID-19 infections. *Proc. Natl. Acad. Sci.* **117**, 27087–27089, DOI: https://doi.org/10.1073/pnas.2010836117 (2020).

21. Schlosser, F. *et al.* Covid-19 lockdown induces disease-mitigating structural changes in mobility networks. *Proc. Natl. Acad. Sci.* **117**, 32883–32890, DOI: https://doi.org/10.1073/pnas.2012326117 (2020).

22. Kissler, S. M. *et al.* Reductions in commuting mobility correlate with geographic differences in SARS-CoV-2 prevalence in New York City. *Nat. Commun.* **11**, 4674, DOI: https://doi.org/10.1038/s41467-020-18271-5 (2021).

23. Correia, S. Reghdfe: Stata module to perform linear or instrumental-variable regression absorbing any number of high-dimensional fixed effects (2019).

24. Athey, S. & Imbens, G. W. Design-based analysis in difference-in-differences settings with staggered adoption. Working Paper 24963, National Bureau of Economic Research (2018). DOI: https://doi.org/10.3386/w24963.

25. Goodman-Bacon, A. Difference-in-differences with variation in treatment timing. Working Paper 25018, National Bureau of Economic Research (2018). DOI: https://doi.org/10.3386/w25018.

26. Isphording, I. E., Lipfert, M. & Pestel, N. School Re-Openings after Summer Breaks in Germany Did Not Increase SARS-CoV-2 Cases. IZA Discussion Papers 13790, Institute of Labor Economics (IZA) (2020).

27. von Bismarck-Osten, C., Borusyak, K. & Schönberg, U. The Role of Schools in Transmission of the SARS-CoV-2 Virus: Quasi-Experimental Evidence from Germany. CReAM Discussion Paper Series 2022, Centre for Research and Analysis of Migration (CReAM), Department of Economics, University College London (2020). https://www.cream-migration.org/publ_uploads/CDP_22_20.pdf.

28. Mangrum, D. & Niekamp, P. JUE Insight: College student travel contributed to local COVID-19 spread. *J. Urban Econon.* **.**, 103311, DOI: https://doi.org/10.1016/j.jue.2020.103311 (2020).

29. Dave, D. M., Friedson, A. I., Matsuzawa, K., Sabia, J. J. & Safford, S. Black lives matter protests and risk avoidance: The case of civil unrest during a pandemic. Working Paper 27408, National Bureau of Economic Research (2020). DOI: https://doi.org/10.3386/w27408.

30. Clarke, D. & Schythe, K. T. Eventdd: Stata module to panel event study models and generate event study plots (2021).
